# Disorder of Biological Quality and Autophagy Process in Bovine Oocytes Exposed to Heat Stress and the Effectiveness of In Vitro Fertilization

**DOI:** 10.3390/ijms241311164

**Published:** 2023-07-06

**Authors:** Marcjanna Wrzecińska, Alicja Kowalczyk, Władysław Kordan, Przemysław Cwynar, Ewa Czerniawska-Piątkowska

**Affiliations:** 1 Department of Ruminant Science, West Pomeranian University of Technology, Klemensa Janickiego 29, 71-270 Szczecin, Poland; marcjanna.wrzecinska@zut.edu.pl (M.W.); ewa.czerniawska-piatkowska@zut.edu.pl (E.C.-P.); 2 Department of Environment Hygiene and Animal Welfare, Wroclaw University of Environmental and Life Sciences, Chelmonskiego 38C, 50-576 Wroclaw, Poland; przemyslaw.cwynar@upwr.edu.pl; 3 Department of Animal Biochemistry and Biotechnology, University of Warmia and Mazury, 10-718 Olsztyn, Poland

**Keywords:** reproduction, bovine, fertilization, oocyte, heat-stress, mitochondrion, autophagy

## Abstract

The main problem in dairy herds is reproductive disorders, which are influenced by many factors, including temperature. Heat stress reduces the quality of oocytes and their maturation through the influence of, e.g., mitochondrial function. Mitochondria are crucial during oocyte maturation as well as the process of fertilization and embryonic development. Disturbances related to high temperature will be increasingly observed due to global warming. In present studies, we have proven that exposure to high temperatures during the cleaving of embryos statistically significantly (at the level of *p* < 0.01) reduces the percentage of oocytes that cleaved and developed into blastocysts eight days after insemination. The study showed the highest percentage of embryos that underwent division in the control group (38.3 °C). The value was 88.10 ± 6.20%, while the lowest was obtained in the study group at 41.0 °C (52.32 ± 8.40%). It was also shown that high temperature has a statistically significant (*p* < 0.01) effect on the percentage of embryos that developed from the one-cell stage to blastocysts. The study showed that exposure to a temperature of 41.0 °C significantly reduced the percentage of embryos that split relative to the control group (38.3 °C; 88.10 ± 6.20%). Moreover, it was noted that the highest tested temperature limits the development of oocytes to the blastocyst stage by 5.00 ± 9.12% compared to controls (33.33 ± 7.10%) and cleaved embryos to blastocysts by 3.52 ± 6.80%; the control was 39.47 ± 5.40%. There was also a highly significant (*p* < 0.0001) effect of temperature on cytoplasmic ROS levels after 6 and 12 h IVM. The highest level of mitochondrial ROS was found in the group of oocytes after 6 h IVM at 41.0 °C and the lowest was found in the control group. In turn, at 41.0 °C after 12 h of IVM, the mitochondrial ROS level had a 2.00 fluorescent ratio, and the lowest in the group was 38.3 °C (1.08). Moreover, with increasing temperature, a decrease in the expression level of both LC3 and SIRT1 protein markers was observed. It was proved that the autophagy process was impaired as a result of high temperature. Understanding of the cellular and molecular responses of oocytes to elevated temperatures will be helpful in the development of heat resistance strategies in dairy cattle.

## 1. Introduction

A crucial problem that has been observed in recent years is the reduction of reproductive efficiency in high-yielding dairy cows. This has a direct impact on dairy farming, its profitability, as well as the dairy industry [1,2]. Dairy cattle are sensitive to changes in environmental conditions caused by climate change, mainly due to their fast growth, intensive metabolism, and productivity [3]. Heat stress decreases feed intake, the efficiency of milk yield, and health status, as well as impairing thermoregulation, which affects animal husbandry [3]. The first symptoms of heat stress are changes in the behavior of cows, which are manifested as shorter lying periods and increased thirst [4]. It is also noted that the reproductive potential of dairy cows may be reduced due to elevated temperatures, which also reduces ovarian and corpus luteum function in summer and follicle growth and development; delays sexual maturation of individuals, extending the age of the first calving; and may also affect the retention of placenta [1,5,6]. Moreover, during heat stress in cows, there is an increase in cortisol secretion, which then interferes with the synthesis of follicle-stimulating and luteinizing hormones. This may result in estrus disorders but also reduces the quality of oocytes and impairs development, growth, maturation, and functioning of dominant and preovulatory follicles [5,7]. Exposure to heat stress in animals can lead to atresia of the ovarian follicles and significantly reduce fertility [3,8]. There is also evidence of the effect of higher temperatures on folliculogenesis and oogenesis. These processes are crucial for the oocyte’s development, maturation, and acquisition of competence, which occurs under the influence of signals and hormones [9].

High temperatures will be recorded more and more often due to global warming. Healthy adult cows maintain a body temperature of 38.4–39.1 °C in thermoneutral zones, i.e., in the range of outdoor temperatures of 4–16 °C [10]. Above these temperature indications, i.e., above 25 °C, an increase in body temperature is observed in cattle, which results in heat stress [8,11,12]. Moreover, heat stress has been shown to disrupt the production of steroid hormones, significantly alter the composition of the follicular fluid, and reduce granulosa cells (GCs), which are essential for developing oocyte competence in cows [1,5]. In addition, high ambient temperature causing heat stress promotes the production of reactive oxygen species (ROS), which can adversely affect the redox balance in granulosa cells [1,13]. Accumulation of ROS in granulosa cells induces apoptosis as well as a decrease in estradiol and progesterone synthesis [9]. During folliculogenesis, a mature and/or Graafian follicle forms from a pool of primary follicles. Primary follicles, composed of an oocyte surrounded by a layer of granulosa cells, are insensitive to heat stress. Therefore, exposure to high temperatures during follicle development may impair oocyte competence [14].

Furthermore, this stress has long-term effects on the oocytes, as it takes two or three estrus cycles to regenerate them from damage caused by high temperatures [9]. Another aspect of heat stress is inducing a body response. Heat shock proteins are chaperone proteins that participate in heat protection at the cellular level. Their expression occurs as the body’s response to temperature changes, which aims to maintain homeostasis [15,16]. Heat-shock proteins 70 and 90 (HSP70, HSP90) mediate regulating various physiological processes, including folliculogenesis, oogenesis, or embryo development. HSPs have been shown to promote cell survival by inhibiting cell apoptosis. However, exposure to strong HS increases cell susceptibility to apoptosis compared to mild exposure [15,17].

The hyperthermia experienced by cows in different cycles reduces the growth of the ovarian follicle, directly affects the reduction of the oocyte pool of ovarian cells, and can also affect the oocyte, which subsequently compromises the ability of the embryo and fetus to develop [5,18,19]. Furthermore, studies have shown that the functionality of mitochondria is a crucial determinant of the development potential of oocytes affecting their quality and, thus, fertilization potential [20,21]. Therefore, any dysfunction or too few mitochondria may affect the abnormal development of oocytes and early embryos [20]. Furthermore, it has been shown that exposure of bovine oocytes to high temperatures may disrupt the function of oocyte mitochondria, causing, among others, DNA fragmentation [22].

The quality of oocytes, i.e., their ability to develop into an embryo, is affected by many factors, including diet, stress, and in vitro maturation (IVM) conditions [23,24]. Moreover, the quality of the oocytes is closely related to the granulosa cells [20]. Studies have shown that the functionality of mitochondria is a crucial determinant of the development potential of oocytes affecting their quality [20,21,25,26]. The oocyte contains an average of 10^5^–10^6^ mitochondria, which participate in oxidative phosphorylation and are the primary source of ATP [20]. ATP levels are linked to oocyte developmental competence, including fertilization potential and subsequent embryonic development. In addition, mitochondrial transport may protect oocytes from aging [26]. Lipid metabolism, an essential substrate for energy generation, also occurs in the mitochondria. In turn, lipid metabolism disorders, including increased amounts of fatty acids and incredibly saturated fatty acids in the oocyte, may inhibit the process of fertilization and embryo development [26]. These organelles also maintain Ca^2+^ ion homeostasis, which is necessary during oocyte IVM and fertilization [27,28]. Another role of the mitochondria is to support the early development of the oocyte and embryo, as glycolysis is limited during oocyte maturation, which is observed until early pre-implantation embryonic development. Therefore, the oocyte’s mitochondria are the primary energy source during the development of the embryo [29,30]. It has been found that dysfunction or reduction in the number of mitochondria in oocytes can affect the abnormal growth of these cells and early embryos [20]. Mitochondria are structured in a manner causing them to be highly sensitive to thermal stress [25,28].

Autophagy is believed to be the primary mechanism regulating the intracellular delivery of dysfunctional proteins and protein aggregates to the lysosome [31], thereby playing a pivotal role in both physiological and pathophysiological contexts [32]. It can be induced by various stressors, ranging from food deprivation to hypoxia or disturbance of homeostasis. Its primary phylogenetically conserved role is as an evolutionary catabolic pathway supporting cellular development, maintenance, and homeostasis [33].

Several studies have shown an association between oocyte quality and mitochondrial function [34]. The number of mitochondria and their function is regulated by the organized processes of mitochondrial biosynthesis and degradation in cells [35]. SIRT1 has been shown to be an essential mitochondrial deacetylase involved in the biological functions of mitochondria [36]. It is also involved in the processes of regulation of autophagy and mitochondrial function in cells related to maintaining the REDOX balance [37]. In turn, LC3 is a protein marker located on the autophagosome membrane [35], which also mediates the antioxidant defense processes of oocytes and may be a helpful marker to determine their competence in response to heat stress [38].

The aim of this study was to assess the impact of heat stress on the in vitro fertilization process (experiment 1) and to evaluate the effects of this stress on mitochondrial and cytoplasmic processes in bovine cells (experiment 2). An additional objective was to determine the effect of heat shock on autophagy processes in oocytes (experiment 3).

## 2. Results

### 2.1. Experiment #1: Influence of Heat-Stress Exposure on In Vitro Fertilization Processes

**Figure 1:**
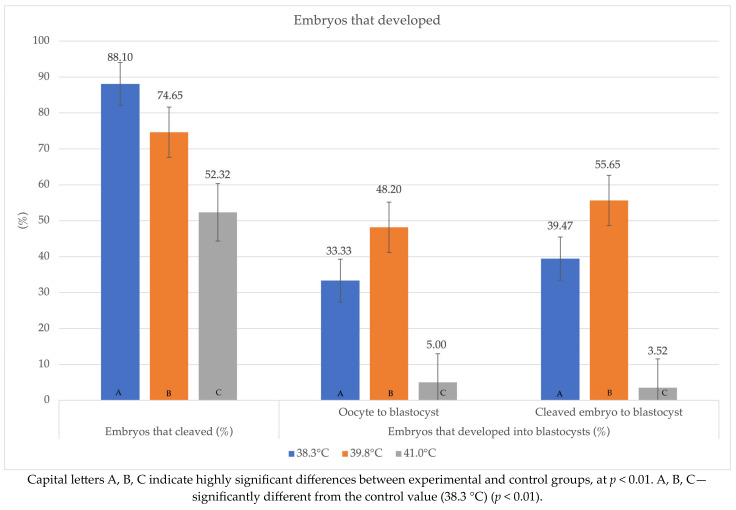
Influence of temperature during fertilization on the percentage of oocytes that cleaved and developed into blastocysts eight days after insemination.

The study showed the highest percentage of embryos that underwent division to be in the control group (38.3 °C). The value was 88.10 ± 6.20%, while the lowest was obtained in the study group at 41.0 °C (52.32 ± 8.40%) (Figure 1). For the study group at 39.8 °C, 74.65 ± 8.90% of the divided embryos were obtained. The highest percentage of oocytes that reached the blastocyst stage was recorded in the group subjected to heat stress at 39.8 °C (48.20 ± 6.60%), while the control group (38.3 °C) was 33.33 ± 7.1% (Figure 1). The lowest percentage of oocytes that reached the blastocyst stage were recorded in the test group at a set temperature of 41.0 °C (5.00 ± 9.12%) (Figure 1). In terms of embryo development to the blastocyst stage, the highest values were obtained in the test group at 39.8 °C (55.65 ± 7.90%); the control group (38.3 °C) had a value of 39.47 ± 5.40%, and the lowest percentage of embryos that reached the blastocyst stage was observed in the group at 41.0 °C (3.52 ± 6.80%) (Figure 1).

All groups differed in a statistically highly significant way (at the level of *p* < 0.01). The influence of temperature was highly influential in each developmental group of germ cells.

**Figure 2:**
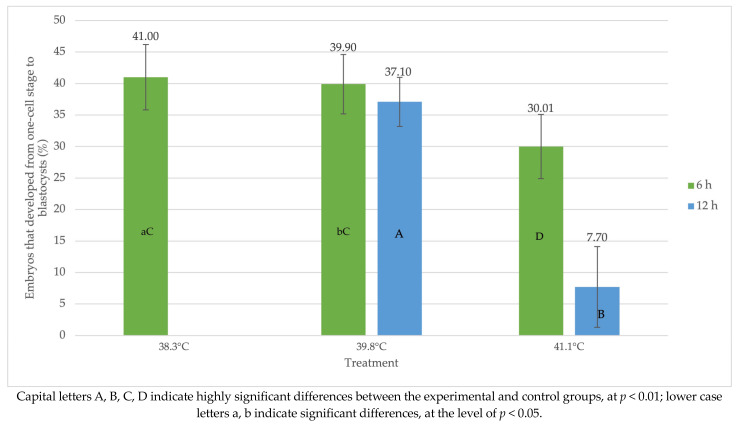
Influence of heat shock at the one-cell stage of development on the percentage of oocytes that cleaved and became blastocysts 8 days after insemination.

Comparing the control group (38.3 °C) to the experimental group (39.8 °C), there was a significant difference in the number of embryos that developed from the unicellular stage to blastocysts (*p* < 0.05) (Figure 2). At the same time, there were highly significant differences (*p* < 0.01) concerning the group exposed to the temperature of 41.0 °C. In the control group, after 6 h, the percentage of embryos that developed from the unicellular stage to blastocysts was 41.0 ± 5.2%, while the lowest rate was in the research group at 41.0 °C (30.1 ± 5.1%). A similar situation concerned the number of embryos developing after 12 h of observation. The difference between the 39.8 °C and 41.0 °C groups was highly statistically significant (*p* < 0.01) and amounted to 37.1 ± 3.9% and 7.7 ± 6.4%, respectively (Figure 2).

### 2.2. Experiment #2: Influence of Heat-Stress Exposure on Mitochondrial and Cytoplasmic Processes

**Figure 3:**
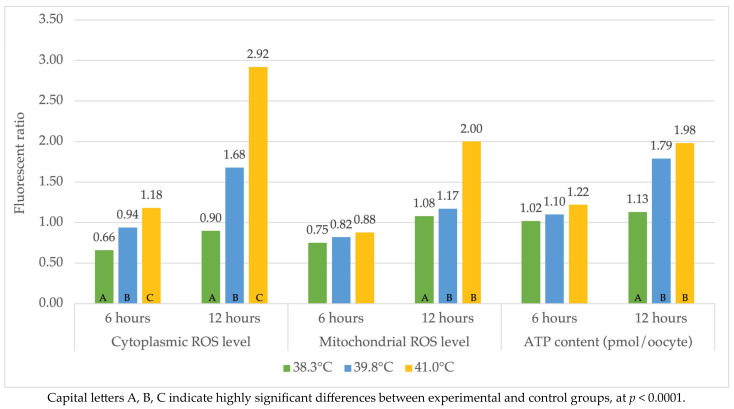
Reactive oxygen species levels and ATP content in heat-stressed oocytes at 6 h and 12 h IVM (during maturation).

The effect of temperature on the level of ROS cytoplasm was highly significant (*p* < 0.0001), both after 6 and 12 h (Figure 3). The highest fluorescent ratio values were obtained after 6 h for the temperature of 41.0 °C (1.18), while the lowest values were recorded in the control group (38.3 °C) and amounted to 0.66. Similar indications were detected after 12 h, where IVM at 41.0 °C was characterized by the highest cytoplasmic ROS content (2.92), and the lowest was obtained at 38.3 °C (0.90) (Figure 3). The highest level of mitochondrial ROS was found in the group of oocytes after 6 h IVM at 41.0 °C (0.88) and the lowest in the control group (0.75) (Figure 3). In turn, at 41.0 °C after 12 h of IVM, the mitochondrial ROS level had a 2.00 fluorescent ratio, and the lowest in the group was 38.3 °C (1.08) (Figure 3). The highest ATP content was recorded in control oocytes maturing in vitro in the research groups after 6 and 12 h IVM (Figure 3). Mitochondrial ROS and ATP levels were not statistically significantly different in the experimental groups (39.8 °C and 41.0 °C) (Figure 3). Statistically highly significant differences were found between the control group (38.3 °C) and the two experimental groups (*p* < 0.0001).

### 2.3. Experiment #3: Analysis of Autophagy Regulation Processes (Expression of SIRT1 and LC3) during Exposure to Heat Stress

**Figure 4:**
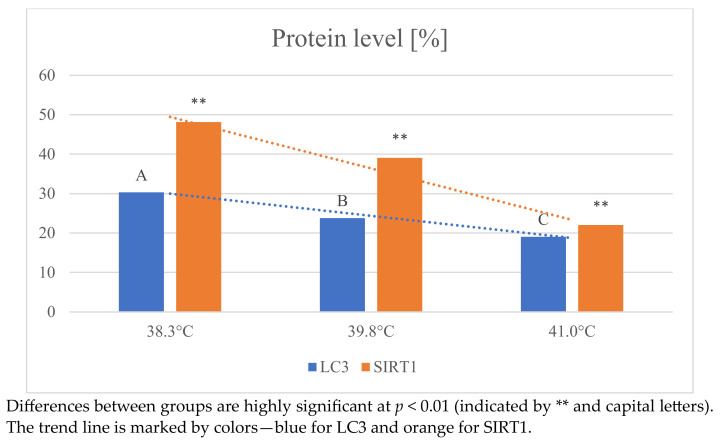
Changes in the expression of LC3 and SIRT1 proteins in oocytes depending on temperature.

Figure 4 shows the effect of heat shock on autophagy processes in oocytes.

With increasing temperature, a decrease in the expression level of both LC3 and SIRT1 protein markers was observed. The expression level of LC3 was highest in the 38.3 °C group (30.30 ± 2.7) and lowest at the highest temperature (19.04 ± 2.1). Identical results were obtained when analyzing changes in SIRT1 level. The highest value was observed at 38.3 °C (48.12 ± 4.6) and the lowest at temperatures above 41 °C (22.00 ± 2.0). The effect of temperature on SIRT1 protein expression was statistically highly significant (*p* < 0.01). The above results clearly indicate that the autophagy process was impaired as a result of high temperature.

## 3. Discussion

The study aimed to assess the impact of heat stress on the process of in vitro fertilization (experiment 1) and to evaluate the effects of this stress on mitochondrial and cytoplasmic processes in bovine cells (experiment 2). An additional objective was to determine the effect of heat shock on autophagy processes in oocytes (experiment 3). Studies show that exposure of cows to heat stress contributes to hyperthermia and disorders within the reproductive system [28,39].

Our research has proven that exposure to heat during in vitro maturation of bovine oocytes impairs their growth and developmental competence. This may confirm that oocytes are sensitive to various factors, including temperature [40]. Furthermore, it has been shown that the environmental conditions prevailing in the summer do not entirely block the developmental competence of oocytes but contribute to the reduction of their development and cleavage and limit the achievement of the blastocyst stage after fertilization. Presented studies showed that the culture of bovine oocytes at stress temperatures of 39.8 °C and 41.0 °C for 6 h and 12 h of in vitro maturation reduced the cleavage capacity from the unicellular stage to the blastocyst stage.

According to current knowledge, the first 12 h of in vitro maturation are crucial in maintaining the developmental competence of oocytes, which then affects their embryonic development ability. During this time, the oocyte goes from the induction phase to the synthesis phase [41]. Then, several changes occur: de novo synthesis of proteins, their accumulation, post-translational modifications, organization of microtubules, or chromatin condensation [41,42,43]. Exposure of oocytes to high temperatures is a significant factor contributing to reduced fertility, including reduced conception rates in inseminated cows [44]. In addition, it reduces the viability of granulosa cells, limiting the production of estradiol necessary during oocyte maturation or embryo development [39]. Oocytes exposed to temperature shock show reduced cleavage capacity and a low capacity to develop into a blastocyst [39]. Heat stress impairs the developmental competence of oocytes at the stage of the germinal vesicle [45]. Reducing cattle’s reproductive potential translates into herds’ production efficiency and generates direct losses for the dairy industry [42,44].

Stamperna et al. [40] reports that exposure of bovine oocytes to high temperatures for as little as 3 h may result in disturbances in the structure of these cells [40]. In another study by Stamperna et al. [41], the effect of short-term exposure of IVM oocytes and embryos to temperature shock was assessed. For this purpose, the cumulus–oocyte complexes matured in vitro for 24 h at 39 °C in the control group and at 41 °C in the test group for 6 h. Cleavage was assessed 48 h after fertilization. Increased temperature to 41 °C for 6 h has been shown to disrupt oocyte maturation and inhibit cleaved blastocyst formation, which translates into a reduction in embryo quality [41]. The results obtained by Stamperna et al. [41] are similar to the results presented. Moreover, the authors of [41] prove that even a short exposure to temperature shock causes disturbances in the genomic regulation of oocyte maturation. It has also been shown that the given exposure time (6 h) to the temperature of 41 °C disturbs the oxidative balance of the oocyte and cumulus cells [41]. Numerous studies [40,41,42,46] show that exposure of oocytes to high temperatures, both in vivo and in vitro, reduces the ability to fertilize and develop the embryo [40,41,42,46]. Another consistent study by Ispada et al. [42] showed that 14 h exposure of IVM oocytes to a temperature of 41 °C reduces the ability of the oocyte to cleave and develop into the blastocyst stage.

In the study, as a result of a temperature shock (39.8 °C and 41.0 °C), an increase in the content of reactive oxygen species in the mitochondria and cytoplasm of oocytes was obtained. Reactive oxygen species damage the genetic material of cells, leading to cell dysfunction and even inducing apoptosis [47]. Reduced function of oocyte mitochondria is observed during exposure to heat stress [42]. The proper functioning of the mitochondria is crucial for maintaining the competence of the oocyte [48]. In cumulus cells, an increase in oxidative metabolism, an energy source, is observed. These processes make up the process of oocyte maturation. Exposure to high temperatures between 18–21 h of IVM has been shown to lead to cytoplasmic disturbances similar to those associated with cell aging [41]. These changes lead to DNA fragmentation, which can directly result in cell apoptosis [42].

Oocytes exposed to high temperatures are prone to higher production of reactive oxygen species, increasing the metabolic activity of the cells and consequently exposing the cells to oxidative stress [49]. Heat stress changes the activity of antioxidant enzymes in the cell, increasing its susceptibility to oxidative damage within the oocyte structures and affecting the development of embryos [42]. High temperatures can damage cellular organelles, including the mitochondrion, limiting their functioning [50]. Mitochondria play an essential role in oocytes. They participate in calcium homeostasis; the production of mitochondrial ATP, which is the primary source of energy, during respiration; the regulation of oxidation and reduction reactions in the cytoplasm; and they participate in oocyte apoptosis [26,28,51]. Thermal shock opens the permeability transition pores, reducing mitochondrial activity in immature and mature oocytes [42,49]. Changes taking place in the mitochondria of oocytes have a direct impact on their developmental abilities [51]. The increase in ROS due to heat stress damages the mitochondria during oocyte maturation, reducing their developmental competence [42,49]. In the conducted study, as a result of a temperature shock (39.8 °C and 41.0 °C), an increase in the content of reactive oxygen species in the mitochondria and cytoplasm of oocytes was observed. Reactive oxygen species damage the genetic material of cells, leading to cell dysfunction and even inducing apoptosis [47]. The proper functioning of the mitochondria is crucial for maintaining the competence of the oocyte [48]. In cumulus cells, an increase in oxidative metabolism, an energy source, is observed. These processes make up the process of oocyte maturation. Exposure to high temperatures between 18–21 h of IVM has been shown to lead to cytoplasmic disturbances similar to those associated with cell aging [41]. These changes lead to DNA fragmentation, which may result in cell apoptosis [42].

Lee et al. [52] experimented on 158 oocytes from Holstein cattle and 123 oocytes from Jersey cows, with which the effect of temperature on the development of female gametes was investigated. The tested temperature was 40.5 °C and 37.5 °C as a control. The study showed that ROS levels differed between Holstein (19.30 ± 0.74) and Jersey (16.46 ± 1.10) oocytes in control temperature. In turn, significant differences were obtained under heat stress (40.5 °C), in which Holstein oocytes indicated a decreased mean of ROS levels (32.20 ± 0.91) compared to Jersey oocytes (20.34 ± 1.07) [52]. Moreover, it suggests that the excessive reactive oxygen species lead to cytoplasmic defects within the oocyte and damage to the genetic material [52]. Properly functioning of the mitochondria is crucial during stressful conditions when the amount of reactive oxygen species increases, contributing to permanent damage. Furthermore, induced oxidative stress causes damage to the mitochondria, which reduces the source of energy and the availability of ATP. These phenomena can hurt the viability of the oocyte [40]. Disorders of mitochondrial respiration, the product of which is energy in the form of ATP, affect ATP levels in the cytoplasm [26]. Elevated levels of ATP in the oocyte cytoplasm are observed when cells are exposed to heat stress [19,53]. This may impair oocyte development, fertilization potential, or subsequent embryonic development [26,51].

The relationship between ROS levels and ATP is not sufficiently understood. Some studies suggest that excessive ROS production in oocytes causes a decrease in intracellular ATP concentration [54], while other data indicate that SIRT may increase ATP levels and thus protect cells from ROS-mediated oxidative damage [37]. SIRT1 is an essential mitochondrial deacetylase that regulates mitochondrial biological functions of mitochondria [36] and is directly related to the regulation of autophagy and mitochondrial function in cells, which can increase the ATP content of cells and protect them from excessive reactive oxygen species (ROS) and oxidative damage [37]. In our study, we showed that excessive ROS production and an increase in ATP concentration were associated with a decrease in SIRT1 and LC3.

Due to the progressive warming of the climate, the reduction of fertility in dairy cows and the decrease in their milk yield may significantly limit the dairy industry.

## 4. Materials and Methods

Considering the fact that this research covers only veterinary procedures, which are not experimental but merely routine breeding activities, it was not necessary to obtain the approval of the local animal ethics committee for this purpose.

### 4.1. Experiment #1: Influence of Heat-Stress Exposure on In Vitro Fertilization Processes

#### 4.1.1. Collected and Matured Cumulus–Oocyte Complexes

Material for the study was collected from 21 cows aged 16 to 24 months. The animals came from the regions of southwestern and western Poland. Bovine cumulus–oocyte complexes (COCs) were collected and matured according to the procedure described by Rispoli (2011) [55] at 38.3 °C for 6 and 12 h (control) or 39.8 and 41.0 °C for 6 and 12 h (heat stress). After 24 h of in vitro maturation (IVM), a subset of control and heat-stressed COCs were denuded of their associated cumulus by vortexing in 0.3% hyaluronidase. Cumulus-free oocytes were lysed in RNA extraction buffer (Thermo Fisher Scientific, Waltham, MA, USA) and then stored at −80 °C (*n* = 80 oocytes per each treatment). The remaining COCs were fertilized in vitro, and embryo culture was collected.

#### 4.1.2. Fertilization (In Vitro)

Bovine ovaries were obtained from a local abattoir located approximately 60 min from the laboratory. Ovaries were transported to the laboratory in 0.9% (*w*/*v*) NaCl at room temperature. The ovaries were sliced, and oocyte–cumulus complexes were collected into a beaker containing oocyte collection medium as previously described [56].

Spermatozoa (fresh, from one male, collected according to Kowalczyk et al. [57]) that had been purified by Percoll gradient centrifugation [58] and suspended in SP-TALP were added to the matured oocytes at a density of approximately 1 × 10^6^ spermatozoa per well. Coculture of spermatozoa and COCs proceeded for 10 h, after which time the putative zygotes were denuded of cumulus cells by vortexing in a 2.0 mL microcentrifuge tube containing 0.5 mL Hepes-TALP for 5 min. The culture media were prepared using recipes described by Parrish et al. [58].

##### Heat Shock Induction

Coculture of spermatozoa and COCs was performed for 6 and 12 h at 38.3, 39.8, and 41.0 °C. After fertilization, putative zygotes were cultured at 38.3 °C for 8 days. The cleavage rate was determined on day 3 after insemination, and development to the blastocyst stage was determined on day 8 after insemination. This experiment was replicated eight times using a total of 200–210 oocytes per treatment. The stage of blastocyst development was assessed by the method previously described by Schrock [56].

##### One-Cell Stage

After the coculture of spermatozoa and COCs was complete (10 h after insemination), putative zygotes were cultured in 50 mL microdrops in groups of 25–30 at 38.3 °C continuously (control group) or were exposed to 39.8 or 41.0 °C for 6 or 12 h (treatment groups). After this time, all embryos were cultured at 38.3 °C for the duration of the culture. Cleavage rate was determined on 72 h after insemination, and development to the blastocyst stage was determined on day 8 [56].

### 4.2. Experiment #2: Influence of Heat-Stress Exposure on Mitochondrial and Cytoplasmic Processes

#### 4.2.1. Reactive Oxygen Species Measurement

Reactive oxygen species (ROS) levels were measured according to the methodology described by Payton et al. [19]. For this purpose, the level of reactive oxygen species was measured after culturing cumulus–oocyte complexes at the germ follicle stage for 6 h at 38.3 (control), 39.8, and 41.0 °C. After 12 h, oocytes were vortexed in 0.3% hyaluronidase to remove cumulus cells, and zona pellucida was removed with 0.5% pronase. The oocytes after this treatment were ready for the assessment of ROS levels in the mitochondria using 60 μM dihydrofluorescein diacetate (DHF; Fluka/Sigma-Aldrich, St. Louis, MO, USA) in HEPES-TL with 1% polyvinylpyrrolidone (HEPES-PVP), and cytoplasmic ROS was assessed with 37 μM diacetate 6-carboxy-2′7′-dichlorodihydrofluorescein, di(acetoxymethyl ester) [DCDHF; Invitrogen/Thermo Fisher Scientific] in HEPES-PVP. Hydrogen peroxide (200 μM; ACS certified; Thermo Fisher Scientific, Waltham, MA, USA) and tert-butyl hydroperoxide (100 μM tertBOOH; Fluka/Sigma-Aldrich) in HEPES-PVP were used as positive controls. Samples were imaged using a Nikon Eclipse TE300 (Nikon Instruments; Melville, NY, USA; 4′,6′-diamidino-2-phenylindole filter: excites at 330–380 nm) in NIS-Elements BR software (version 3.0; Nikon).

#### 4.2.2. Measurement of ATP Content

Cumulus–oocyte complexes were matured for 12 h at 38.3, 39.8, and 41.0 °C. After a total of 24 h, cumulus cells and zona pellucida were removed from a subset of oocytes using the same methods as described above. Denuded oocytes were frozen at −80 °C for later analysis of ATP. ATP content was assessed in individual embryos using the ATP Determination kit (Invitrogen™/Life Technologies/Thermo Fisher Scientific) [59]. A six-point standard curve (0–5 pmol) was considered in each test series. Standard curves were generated, and the ATP content was calculated using the formula obtained from the linear regression of the standard curve.

### 4.3. Experiment #3: Analysis of Autophagy Regulation Processes (Expression of SIRT1 and LC3) during Exposure to Heat Stress

#### SIRT1 and LC3 Expression Analysis

After the 6 and 12 h oocyte culture period in the IVM medium, immunofluorescence analysis was performed [60]. Briefly, after removal of the pellucid zone by acidic Tyrod solution (Sigma-Aldrich, Merck, Saint Louis, MI, USA), oocytes were fixed and flushed with 4% paraformaldehyde with 0.1% Triton X-100 in phosphate-buffered saline (PBS, Sigma-Aldrich, Merck, USA) for 20 min at 25 °C and then washed with 0.3% Triton X-100 in PBS for 5 min. The oocytes were then blocked in 10% bovine serum albumin (BSA, Sigma-Aldrich, Merck, USA)/PBS drops for 30 min. Finally, they were incubated with a primary antibody containing anti-LC3 and anti-Sirt1 [rabbit polyclonal, 1:100 (Abcam, Waltham, MA, USA)] in 2% BSA/PBS at 4 °C overnight. The next day, the oocytes were washed three times in 2% BSA/PBS and incubated with goat anti-rabbit IgG fluorescein conjugated (1:200; Abcam, USA) as a secondary antibody at 37 °C for 40 min. After washing three times with PBS, oocytes were mounted on slides using an anti-fading reagent containing 6-diamidino-2-phenylindole (DAPI, Sigma-Aldrich, USA). SIRT1 and LC3 expression was assessed using a fluorescence microscope (Levenhuk MED PRO 600 Fluo, USA, Levenhuk Inc. (Tampa, FL, USA): 928 E 124th Ave. Ste D, Tampa, FL, USA) at an excitation wavelength of 488.

### 4.4. Statistical Analyses

Analyzing the effect of temperature on protein levels, a one-way analysis of variance with the LSD (Least Significant Difference) test was used [61]. Significant differences (*p* < 0.01) are indicated by different capital letters and two asterisks (**; A,B,C).

## 5. Conclusions

The results obtained in this study confirmed that exposure of maturing oocytes to high temperatures has a devastating effect on their developmental competence and subsequent blastocyst development. In addition, exposure to heat changes mitochondrial function by affecting energy levels (ATP) and redox potential (ROS levels). Higher ATP synthesis in oocytes exposed to temperature shock may confirm that the mitochondria of these oocytes had a reduced mitochondrial membrane potential. Moreover, with increasing temperature, a decrease in the expression level of both LC3 and SIRT1 protein markers was observed.

Heat stress reduces the reproductive performance of cattle, so a thorough understanding of the cellular and molecular responses of oocytes to elevated temperatures will be helpful in the development of heat resistance strategies in dairy cattle.

## Data Availability

The data presented in this study are available on request from the corresponding author.

## References

[1] Ho K.T., Balboula A.Z., Homma K., Takanari J., Bai H., Kawahara M., Thi Kim Nguyen K., Takahashi M. (2023). Synergistic Effect of Standardized Extract of Asparagus Officinalis Stem and Heat Shock on Progesterone Synthesis with Lipid Droplets and Mitochondrial Function in Bovine Granulosa Cells. J. Steroid Biochem. Mol. Biol..

[2] Wiebke M., Pieper L., Gürler H., Janowitz U., Jung M., Schulze M. (2023). Effect of Using Liquid Semen on Fertility in German Holstein Friesian Dairy Cattle: A Randomized Controlled Clinical Trial. Theriogenology.

[3] Zheng Y., Xie T., Li S., Wang W., Wang Y., Cao Z., Yang H. (2022). Effects of Selenium as a Dietary Source on Performance, Inflammation, Cell Damage, and Reproduction of Livestock Induced by Heat Stress: A Review. Front. Immunol..

[4] Frigeri K.D.M., Deniz M., Damasceno F.A., Barbari M., Herbut P., Vieira F.M.C. (2023). Effect of Heat Stress on the Behavior of Lactating Cows Housed in Compost Barns: A Systematic Review. Appl. Sci..

[5] Gad A., Joyce K., Menjivar N.G., Heredia D., Rojas C.S., Tesfaye D., Gonella-Diaza A. (2023). Extracellular Vesicle-MicroRNAs Mediated Response of Bovine Ovaries to Environmental Heat Stress. J. Ovarian Res..

[6] Koester L.R., Hayman K., Anderson C.J., Tibbs-Cortes B.W., Daniels K.M., Seggerman F.M., Gorden P.J., Lyte M., Schmitz-Esser S. (2023). Influence of a Sodium-Saccharin Sweetener on the Rumen Content and Rumen Epithelium Microbiota in Dairy Cattle during Heat Stress. J. Anim. Sci..

[7] Çizmeci S.Ü., Dinç D.A., Yesilkaya O.F., Çiftçi M.F., Takcı A., Bucak M.N. (2022). Effects of Heat-Stress on Oocyte Number and Quality and In Vitro Embryo Production in Holstein Heifers. Acta Scientiae. Vet..

[8] Gupta S., Sharma A., Joy A., Dunshea F.R., Chauhan S.S. (2022). The Impact of Heat Stress on Immune Status of Dairy Cattle and Strategies to Ameliorate the Negative Effects. Animals.

[9] Khan A., Khan M.Z., Umer S., Khan I.M., Xu H., Zhu H., Wang Y. (2020). Cellular and Molecular Adaptation of Bovine Granulosa Cells and Oocytes under Heat Stress. Animals.

[10] Havelka Z., Kunes R., Kononets Y., Stokes J.E., Smutny L., Olsan P., Kresan J., Stehlik R., Bartos P., Xiao M. (2022). Technology of Microclimate Regulation in Organic and Energy-Sustainable Livestock Production. Agriculture.

[11] Miętkiewska K., Kordowitzki P., Pareek C.S. (2022). Effects of Heat Stress on Bovine Oocytes and Early Embryonic Development—An Update. Cells.

[12] Wrzecińska M., Czerniawska-Piątkowska E., Kowalczyk A. (2021). The Impact of Stress and Selected Environmental Factors on Cows’ Reproduction. J. Appl. Anim. Res..

[13] Zeng H.-F., Xu J., Wang X.-L., Li S.-J., Han Z.-Y. (2022). Nicotinamide Mononucleotide Alleviates Heat Stress-Induced Oxidative Stress and Apoptosis in BMECs through Reducing Mitochondrial Damage and Endoplasmic Reticulum Stress. Ecotoxicol. Environ. Saf..

[14] Ram S., Awasthi M.K., Khan J.R., Kaiser P., Singh N. (2023). Effect of Stress on Ovarian Follicular Activity in Postpartum Sahiwal Cows during Hot-Humid Season. Ind. J. Vet. Sci. Biotech..

[15] Pöhland R., Souza-Cácares M.B., Datta T.K., Vanselow J., Martins M.I.M., da Silva W.A.L., Cardoso C.J.T., Melo-Sterza F. (2020). de A. Influence of Long-Term Thermal Stress on the in Vitro Maturation on Embryo Development and Heat Shock Protein Abundance in Zebu Cattle. Anim. Reprod..

[16] Stamperna K., Giannoulis T., Dovolou E., Kalemkeridou M., Nanas I., Dadouli K., Moutou K., Mamuris Z., Amiridis G.S. (2021). Heat Shock Protein 70 Improves In Vitro Embryo Yield and Quality from Heat Stressed Bovine Oocytes. Animals.

[17] Yang Y.-W., Chen L., Yang C.-X. (2019). Heat Shock Protein 90 and Reproduction in Female Animals: Ovary, Oocyte and Early Embryo. Heat Shock Protein 90 in Human Diseases and Disorders.

[18] Ghaffari M.H. (2022). Developmental Programming: Prenatal and Postnatal Consequences of Hyperthermia in Dairy Cows and Calves. Domest. Anim. Endocrinol..

[19] Payton R.R., Rispoli L.A., Nagle K.A., Gondro C., Saxton A.M., Voy B.H., Edwards J.L. (2018). Mitochondrial-Related Consequences of Heat Stress Exposure during Bovine Oocyte Maturation Persist in Early Embryo Development. J. Reprod. Dev..

[20] Shen Q., Liu Y., Li H., Zhang L. (2021). Effect of Mitophagy in Oocytes and Granulosa Cells on Oocyte Quality †. Biol. Reprod..

[21] Zhao S., Heng N., Wang H., Wang H., Zhang H., Gong J., Hu Z., Zhu H. (2022). Mitofusins: From Mitochondria to Fertility. Cell. Mol. Life Sci..

[22] Laporta J., Ferreira F.C., Ouellet V., Dado-Senn B., Almeida A.K., De Vries A., Dahl G.E. (2020). Late-Gestation Heat Stress Impairs Daughter and Granddaughter Lifetime Performance. J. Dairy Sci..

[23] Azari-Dolatabad N., Benedetti C., Velez D.A., Montoro A.F., Sadeghi H., Residiwati G., Leroy J.L.M.R., Van Soom A., Pascottini O.B. (2023). Oocyte Developmental Capacity Is Influenced by Intrinsic Ovarian Factors in a Bovine Model for Individual Embryo Production. Anim. Reprod. Sci..

[24] El-Sheikh M., Mesalam A., Khalil A.A.K., Idrees M., Ahn M.-J., Mesalam A.A., Kong I.-K. (2023). Downregulation of PI3K/AKT/MTOR Pathway in Juglone-Treated Bovine Oocytes. Antioxidants.

[25] Roth Z. (2021). Heat Stress Reduces Maturation and Developmental Capacity in Bovine Oocytes. Reprod. Fertil. Dev..

[26] Zheng L., Luo Y., Zhou D., Liu H., Zhou G., Meng L., Hou Y., Liu C., Li J., Fu X. (2023). Leonurine Improves Bovine Oocyte Maturation and Subsequent Embryonic Development by Reducing Oxidative Stress and Improving Mitochondrial Function. Theriogenology.

[27] Jegal H.-G., Park H.-J., Kim J.-W., Yang S.-G., Kim M.-J., Koo D.-B. (2020). Ruthenium Red Improves Blastocyst Developmental Competence by Regulating Mitochondrial Ca2+and Mitochondrial Functions in Fertilized Porcine Oocytes in Vitro. J. Reprod. Dev..

[28] Shi X., Jin X., Lin J., Sun L., Liu X., Zhang T., Wang M., Yue S., Zhou J. (2022). Idebenone Relieves the Damage of Heat Stress on the Maturation and Developmental Competence of Porcine Oocytes. Reprod. Domest. Anim..

[29] Babayev E., Seli E. (2015). Oocyte Mitochondrial Function and Reproduction. Curr. Opin. Obstet. Gynecol..

[30] De Andrade Melo-Sterza F., Poehland R. (2021). Lipid Metabolism in Bovine Oocytes and Early Embryos under In Vivo, In Vitro, and Stress Conditions. Int. J. Mol. Sci..

[31] Lilienbaum A. (2013). Relationship between the Proteasomal System and Autophagy. Int. J. Biochem. Mol. Biol..

[32] Levine B., Kroemer G. (2008). Autophagy in the Pathogenesis of Disease. Cell.

[33] Farhan H., Kundu M., Ferro-Novick S. (2017). The Link between Autophagy and Secretion: A Story of Multitasking Proteins. MBoC.

[34] Iwata H., Goto H., Tanaka H., Sakaguchi Y., Kimura K., Kuwayama T., Monji Y. (2011). Effect of Maternal Age on Mitochondrial DNA Copy Number, ATP Content and IVF Outcome of Bovine Oocytes. Reprod. Fertil. Dev..

[35] Sugiyama M., Kawahara-Miki R., Kawana H., Shirasuna K., Kuwayama T., Iwata H. (2015). Resveratrol-Induced Mitochondrial Synthesis and Autophagy in Oocytes Derived from Early Antral Follicles of Aged Cows. J. Reprod. Dev..

[36] Pi H., Xu S., Reiter R.J., Guo P., Zhang L., Li Y., Li M., Cao Z., Tian L., Xie J. (2015). SIRT3-SOD2-MROS-Dependent Autophagy in Cadmium-Induced Hepatotoxicity and Salvage by Melatonin. Autophagy.

[37] Ahn B.-H., Kim H.-S., Song S., Lee I.H., Liu J., Vassilopoulos A., Deng C.-X., Finkel T. (2008). A Role for the Mitochondrial Deacetylase Sirt3 in Regulating Energy Homeostasis. Proc. Natl. Acad. Sci. USA.

[38] Yang Q., Dai S., Luo X., Zhu J., Li F., Liu J., Yao G., Sun Y. (2018). Melatonin Attenuates Postovulatory Oocyte Dysfunction by Regulating SIRT1 Expression. REP.

[39] Rispoli L.A., Lawrence J.L., Payton R.R., Saxton A.M., Schrock G.E., Schrick F.N., Middlebrooks B.W., Dunlap J.R., Parrish J.J., Edwards J.L. (2011). Disparate Consequences of Heat Stress Exposure during Meiotic Maturation: Embryo Development after Chemical Activation vs Fertilization of Bovine Oocytes. Reproduction.

[40] Schrock G.E., Saxton A.M., Schrick F.N., Edwards J.L. (2007). Early In Vitro Fertilization Improves Development of Bovine Ova Heat Stressed During In Vitro Maturation. J. Dairy Sci..

[41] Kowalczyk A., Gałęska E., Czerniawska-Piątkowska E., Szul A., Hebda L. (2021). The Impact of Regular Sperm Donation on Bulls’ Seminal Plasma Hormonal Profile and Phantom Response. Sci. Rep..

[42] Parrish J.J., Susko-Parrish J.L., Leibfried-Rutledge M.L., Critser E.S., Eyestone W.H., First N.L. (1986). Bovine in Vitro Fertilization with Frozen-Thawed Semen. Theriogenology.

[43] Park S.-H., Yu I.-J. (2013). Effect of Dibutyryl Cyclic Adenosine Monophosphate on Reactive Oxygen Species and Glutathione of Porcine Oocytes, Apoptosis of Cumulus Cells, and Embryonic Development. Zygote.

[44] Tatone C., Amicarelli F., Carbone M.C., Monteleone P., Caserta D., Marci R., Artini P.G., Piomboni P., Focarelli R. (2008). Cellular and Molecular Aspects of Ovarian Follicle Ageing. Hum. Reprod. Update.

[45] (2008). Least Significant Difference Test Least Significant Difference Test. The Concise Encyclopedia of Statistics.

[46] Berling F., Castro F.C.d., Oliveira A.C.d.S. (2022). Influência Do Estresse Calórico Na Produção in Vitro de Oócitos e Embriões de Vacas Holandesas de Alta Produtividade. Ciênc. Anim. Bras..

[47] Stamperna K., Dovolou E., Giannoulis T., Kalemkeridou M., Nanas I., Dadouli K., Moutou K., Mamuris Z., Amiridis G.S. (2021). Developmental Competence of Heat Stressed Oocytes from Holstein and Limousine Cows Matured in Vitro. Reprod. Dom. Anim..

[48] Stamperna K., Giannoulis T., Nanas I., Kalemkeridou M., Dadouli K., Moutou K., Amiridis G.S., Dovolou E. (2020). Short Term Temperature Elevation during IVM Affects Embryo Yield and Alters Gene Expression Pattern in Oocytes, Cumulus Cells and Blastocysts in Cattle. Theriogenology.

[49] Ispada J., Rodrigues T.A., Risolia P.H.B., Lima R.S., Gonçalves D.R., Rettori D., Nichi M., Feitosa W.B., Paula-Lopes F.F. (2018). Astaxanthin Counteracts the Effects of Heat Shock on the Maturation of Bovine Oocytes. Reprod. Fertil. Dev..

[50] Rodrigues T.A., Tuna K.M., Alli A.A., Tribulo P., Hansen P.J., Koh J., Paula-Lopes F.F. (2019). Follicular Fluid Exosomes Act on the Bovine Oocyte to Improve Oocyte Competence to Support Development and Survival to Heat Shock. Reprod. Fertil. Dev..

[51] Nishisozu T., Singh J., Abe A., Okamura K., Dochi O. (2023). Effects of the Temperature-Humidity Index on Conception Rates in Holstein Heifers and Cows Receiving in Vitro-Produced Japanese Black Cattleembryos. J. Reprod. Dev..

[52] Kawano K., Sakaguchi K., Madalitso C., Ninpetch N., Kobayashi S., Furukawa E., Yanagawa Y., Katagiri S. (2022). Effect of Heat Exposure on the Growth and Developmental Competence of Bovine Oocytes Derived from Early Antral Follicles. Sci. Rep..

[53] Diaz F.A., Gutierrez-Castillo E.J., Foster B.A., Hardin P.T., Bondioli K.R., Jiang Z. (2021). Evaluation of Seasonal Heat Stress on Transcriptomic Profiles and Global DNA Methylation of Bovine Oocytes. Front. Genet..

[54] Báez F., López Darriulat R., Rodríguez-Osorio N., Viñoles C. (2022). Effect of Season on Germinal Vesicle Stage, Quality, and Subsequent in Vitro Developmental Competence in Bovine Cumulus-Oocyte Complexes. J. Therm. Biol..

[55] Ascari I.J., Alves N.G., Jasmin J., Lima R.R., Quintão C.C.R., Oberlender G., Moraes E.A., Camargo L.S.A. (2017). Addition of Insulin-like Growth Factor I to the Maturation Medium of Bovine Oocytes Subjected to Heat Shock: Effects on the Production of Reactive Oxygen Species, Mitochondrial Activity and Oocyte Competence. Domest. Anim. Endocrinol..

[56] Residiwati G., Azari-Dolatabad N., Tuska H.S.A., Sidi S., Van Damme P., Benedetti C., Montoro A.F., Luceno N.L., Budiono, Pavani K.C. (2021). Effect of Lycopene Supplementation to Bovine Oocytes Exposed to Heat Shock during in Vitro Maturation. Theriogenology.

[57] Abdelnour S.A., Yang C.-Y., Swelum A.A., Abd El-Hack M.E., Khafaga A.F., Abdo M., Shang J.-H., Lu Y.-Q. (2020). Molecular, Functional, and Cellular Alterations of Oocytes and Cumulus Cells Induced by Heat Stress and Shock in Animals. Environ. Sci. Pollut. Res..

[58] Roth Z. (2018). Symposium Review: Reduction in Oocyte Developmental Competence by Stress Is Associated with Alterations in Mitochondrial Function. J. Dairy Sci..

[59] Lee J., Kim D.-S., Choi I., Kim D., Son J., Jeon E., Jung D., Han M., Ha S., Hwang S. (2022). Effects of Heat Stress on Conception in Holstein and Jersey Cattle and Oocyte Maturation in Vitro. J. Anim. Sci. Technol..

[60] de Aguiar L.H., Hyde K.A., Pedroza G.H., Denicol A.C. (2020). Heat Stress Impairs In Vitro Development of Preantral Follicles of Cattle. Anim. Reprod. Sci..

[61] Liang S., Guo J., Choi J.-W., Kim N.-H., Cui X.-S. (2017). Effect and Possible Mechanisms of Melatonin Treatment on the Quality and Developmental Potential of Aged Bovine Oocytes. Reprod. Fertil. Dev..

